# Comparison of downstream processing methods in purification of highly active laccase

**DOI:** 10.1007/s00449-019-02160-3

**Published:** 2019-06-15

**Authors:** Anna Antecka, Michał Blatkiewicz, Tomasz Boruta, Andrzej Górak, Stanisław Ledakowicz

**Affiliations:** Faculty of Process and Environmental Engineering, Lodz University of Technology, ul. Wolczanska 213, 90-924 Lodz, Poland

**Keywords:** Laccase, *Cerrena unicolor*, Ultrafiltration, Aqueous two-phase extraction, Foam fractionation

## Abstract

Laccases have received the attention of researchers in the last few decades due to their ability to degrade phenolic and lignin-related compounds. This study aimed at obtaining the highest possible laccase activity and evaluating the methods of its purification. The crude laccase from bioreactor cultivation of *Cerrena unicolor* fungus was purified using ultrafiltration, aqueous two-phase extraction (ATPE) and foam fractionation (FF), which allowed for the assessment of these three downstream processing (DSP) methods. The repeated fed-batch cultivation mode applied for the enzyme production resulted in a high laccase specific activity in fermentation broth of 204.1 U/mg. The use of a specially constructed spin filter inside the bioreactor enabled the integration of enzyme biosynthesis and biomass filtration in one apparatus. Other methods of laccase concentration and purification, namely ATPE and FF, proved to be useful for laccase separation; however, the efficiency of FF was rather low (recovery yield of 24.9% and purification fold of 1.4). Surprisingly, the recovery yield after ATPE in a PEG 6000-phosphate system in salt phase was higher (97.4%) than after two-step ultrafiltration (73.7%). Furthermore, it was demonstrated that a simple, two-step purification procedure resulted in separation of two laccase isoforms with specific activity of 2349 and 3374 U/mg. All in all, a compact integrated system for the production, concentration and separation of fungal laccases was proposed.

## Introduction

The selection of the appropriate method for biomolecules purification is crucial in the evaluation of the economic performance of the whole upstream and downstream processes. In the enzyme production processes, biosynthesis of the desired product is typically followed by several concentration and separation stages (downstream processing, DSP), which are known to be costly and time consuming. However, the viability of those methods depends on the proper choice of the purification techniques and process conditions. It holds also for laccases (EC 1.10.3.2, polyphenol oxidases) that are enzymes belonging to the group of oxidoreductases [1]. They occur in many species of fungi, in higher plants and bacteria [2]. Laccases are characterized by their specific catalytic properties to transfer electrons from donors to oxygen. The ability to oxidize various organic compounds, including phenols, aromatic amines and their derivatives, makes these enzymes particularly relevant for industrial and environmental applications [3, 4]. Although laccases are considered relatively active enzymes in their natural state, their concentration and activity in fermentation does not meet the specifications, expected in industrial applications [5]. Therefore, effective purification methods of laccases solution are necessary.

DSP of laccases, which generally determines up to 80% of the production cost of biomolecules [6], has not been yet satisfactorily investigated. In literature, there are several publications dealing with the purification of laccases from culture supernatants of various microorganisms. The most commonly applied technique is chromatography, which involves different mechanisms of ion exchange, affinity and hydrophobic interactions [7]. Additionally, other methods like microfiltration, ultrafiltration and acetone precipitation are used [8]. They provide highly concentrated products of relatively high purity. However, they suffer from low capacity, high operating costs and long operating time. As a result, the manufacturing process as a whole cannot be regarded as cost effective. Therefore, industrial production of laccases calls for cheaper and more effective DSP methods. Recently, one of the most economical methods for biomolecule recovery is aqueous two-phase extraction (ATPE), which involves two immiscible water phases, with water content usually between 70 and 90% [9, 10]. They can be formed when two mutually immiscible polymers, or a polymer with a salt, an alcohol and a salt, an ionic liquid and a salt, or two surfactants solutions are mixed together [11]. ATPSs have several advantages in the context of protein purification, since they significantly decrease the risk of denaturation [12]. Moreover, the surface tension between phases is low, which leads to the increased rate of mass transfer [13]. ATPE has also been successfully applied for laccase purification and is currently considered as a promising alternative to traditional purification methods. Silvério et al. [14] investigated a few different systems for purification of *Trametes versicolor* laccase and received partitioning coefficients up to *K* = 3.68. Ratanapongleka and Phetsom [9] examined partitioning of *Lentinus polychrous* laccase in PEG-phosphate systems with partitioning coefficients reaching *K* = 66.2. Prinz et al. [15] investigated *Pleurotus sapidus* laccase partitioning in PEG-phosphate system. Blatkiewicz et al. [16] demonstrated that both PEG 400 and PEG 6000 systems allowed for a very strong concentration of *Cerrena unicolor* laccase, with yields exceeding 0.85 and 0.9, respectively. Interestingly, the enzyme concentrated in different phases, with PEG 400 in a polymer and in PEG 6000 in a salt phase. Therefore, applying PEG 6000 was considered a better alternative due to the fact that salt phase is less problematic in terms of further purification.

Another promising DSP method is foam fractionation, which belongs to bubble separation technologies [17]. Foam fractionation occurs when a liquid phase is being continuously fed with dispersed gas stream and forms a foam phase. Chemical compounds which show affinity to gas–liquid interface migrate towards the foam and leave the bulk liquid phase. The foam can be collected and collapsed, as a result a new liquid phase, containing concentrated product, is formed. The first application of the method for protein purification was conducted in 1940 by Ostwald and Mischke for separation of products from yeast fermentation broth [18]. Various parameter studies have been performed to optimize systems for foam fractionation [19–21], but the results show that each system requires independent study due to many, often mutually dependent factors, such as column size, initial protein concentration, pH, competing proteins, detergent concentration, liquid and gas flow rates. Different effects due to feeding point change in the column have also been observed [22]. Although foam fractionation has been recently rediscovered as a DSP method, its application towards laccase purification has not been widely investigated yet. Linke et al. [23] examined the influence of pH on the effectiveness of foam fractionation of laccase from *Trametes* sp., with activity recovery up to 94% in the foamate, and activity losses no greater than 2%. Gerken et al. [24] attempted continuous foam fractionation of laccase C from the fermentation broth, focusing on the feed position of the crude supernatant in the column. They report recoveries up to 70.2% with the feed positioned at the top of the column. Our group focused mainly on laccase isolated from *Cerrena unicolor* and determined that the best activity partitioning coefficients between foamate and retentate was almost 200 with the yield reaching 50% for pH 7.5 and the concentration of cationic surfactant cetrimonium bromide (CTAB) equal to 0.5 mM [25]. Moreover, it was proved that the source of the enzyme is important in terms of partitioning efficiency, as markedly different experimental results were obtained for laccases derived from two distinct fungal species, namely *C. unicolor* and *Pleurotus sapidus* [25].

Taking the above into account, the purpose of this research was to study and integrate the up- and downstream processing of laccase from *Cerrena unicolor* to obtain a highly active enzymatic product. The core of the present work was to prove if the aqueous two-phase extraction and foam fractionation can be used as the purification method of fermentation broth as an intermediate step between fermentation and ultrafiltration and/or chromatographic separation.

## Materials and methods

### Culture cultivation and enzyme production

The *Cerrena unicolor* (Bull. ex Fr.) Murr. strain 137 used in this study was obtained from the culture collection of the Department of Biochemistry, Maria Curie-Sklodowska University, Lublin, Poland. Stock cultures were maintained on 2% malt extract agar (MEA) at 4 °C, and inoculation material was precultivated on MEA plates at 25 °C for 10–14 days. The biosynthesis of laccase was performed in a computer-controlled BIOSTAT ED bioreactor (Sartorius, Germany) of the total working volume of 15 L. The Lindeberg–Holm medium was prepared according to Janusz et al. [26] with glucose and asparagine as the main source of carbon and nitrogen, respectively. The medium was supplemented with Cu^2+^ source, without the use of other inductors. Liquid medium was thermally sterilized in a bioreactor and then inoculated with 240 mL of fungal mycelium from overgrown MEA plates homogenized in sterile water (3 plates in 240 mL of water). Mycelium was homogenized with the use of IKA homogenizer at 8000 rpm and transferred into bioreactor under aseptic conditions. The culture was performed at a constant temperature of 28 °C for 10 days. During the process, the medium was aerated at 2 Ndm^3^ per minute and stirred using a paddle stirrer at 200 rpm. The bioreactor was equipped with a set of sensors for process control, such as: pH and oxygen electrodes, pressure and temperature indicators. Additionally, it was equipped with a special rotary filter type Spinfilter (B. Braun, Germany) installed inside the bioreactor for continuous, sterile separation of biomass and fermentation broth containing laccase [27]. The filter material is made of a multilayer stainless steel woven wire cloth, filtration area is equal to 300 cm^2^ and the mesh diameter is 20 µm. The cultivations were carried out in a repeated fed-batch mode with a periodic addition of the substrate and simultaneous collection of the culture media with the enzyme. Every second day starting from day 5th on, a new portion of the medium was added in the amount of 3 L, simultaneously the same amount of culture liquid without biomass was withdrawn from the bioreactor with the use of a spin filter. Additionally, culture broth samples of 50 mL were collected every day, once per day from the bioreactor under aseptic conditions to measure substrate and product concentrations.

### Purification of laccase

The crude liquid containing mycelia was first filtered through paper filters grade 389 (Munktell) to separate residual mycelia (solids). Subsequently, the obtained filtrate, culture liquid, was purified using three complementary methods.

#### Aqueous two-phase extraction (ATPE)

Extraction in an aqueous two-phase system consisted of polyethylene glycol of molecular weight of 6000 and phosphate buffer solution (pH 7). The extraction experiments were performed in specially designed extraction vessels, similar to the ones presented by Prinz et al. [15]. The vessels consisted of mixing flasks combined with burettes of approximately 20 mL volume. Mixture components, in the form of stock solutions and culture liquid, were prepared according to procedure described by Blatkiewicz et al. [16] and the following proportion was applied: 4.8 g of PEG 6000, 3.4 g KH_2_PO_4_ and 6.8 g of culture liquid. The mixing was carried out in a thermostated incubator equipped with a magnetic stirrer at the temperature set to 25 °C. The samples were stirred for an hour at 300 rpm to achieve phase equilibrium between the phases. After mixing, the flasks were carefully flipped upside down to introduce the mixture into the burette part. Next, the vessels were stored in this position at 25 °C for 24 h to separate the phases. Finally, the flasks were removed from the tank, and the phases were collected into separate vessels and weighed. Samples of each phase were taken, and their enzymatic activities were measured. The extraction experiment was performed in six replications.

#### Foam fractionation (FF)

**Fig. 1:**
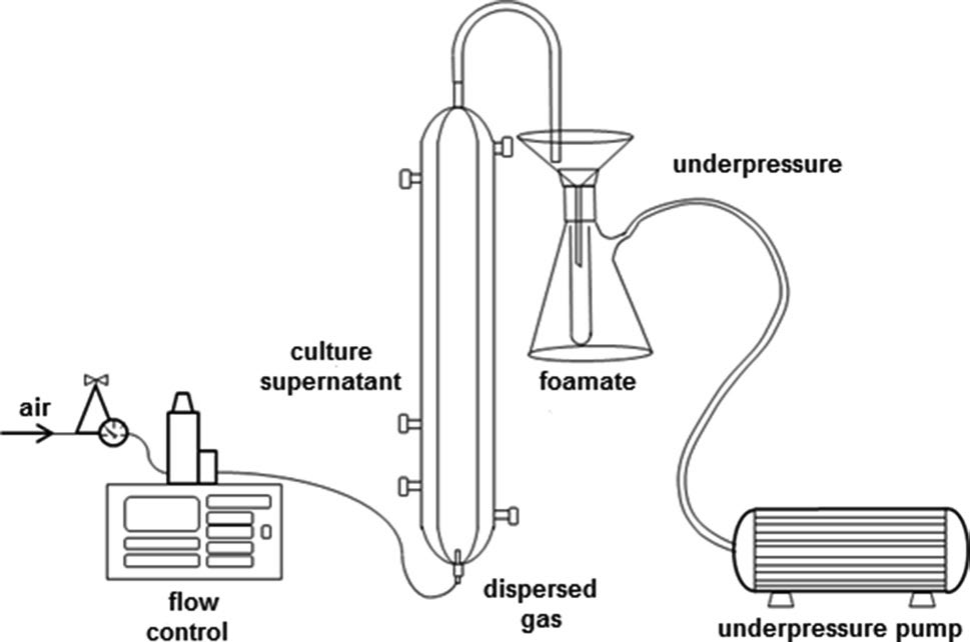
Foam fractionation equipment setup Reproduced from Blatkiewicz et al. [25] with permission granted from Chemical & Process Engineering, De Gruyter

Foam fractionation was performed according to Blatkiewicz et al. [25]. The experimental setup (Fig. 1) consisted of a glass column of 62 cm in length and inner diameter of 3 cm, a foam collector, a Büchner flask connected to a pump producing low under pressure, a compressed air distributor equipped with a reducing valve set to 2 bar and an electronic valve for flux control.

The column was equipped with a porous glass disperser at the bottom. Compressed air flowed to the disperser through an electronic valve which set the airflow to 2.4 L/h. After fixing the airflow, 100 mL of the culture liquid supplemented with the surfactant 0.5 mM cetrimonium bromide (CTAB) was poured into the column, the pH of the mixture was adjusted to 7 and the foaming started. A foam collector was installed at the top of the column which directed the foam into a Büchner flask equipped with a funnel to break the foam down. Experiment was carried out until the foam was no longer able to reach the top of the column before collapsing. At this point, the experiment was terminated and volumes of the foamate (the collapsed foam) and the retentate (the liquid remaining in the column after the process) were measured. Samples of each phase were drawn and their enzymatic activities were measured. All the conducted experiments were performed at room temperature. The experiment was performed in triplicate.

#### Ultrafiltration

As the third method, conventional ultrafiltration was used to concentrate the laccase. In this case, the culture liquid was passed through two steps of ultrafiltration/diafiltration using a Vivaflow 50 PES membrane (Sartorius, United Kingdom) with a cut-off of 10 and 30 kDa and equilibrated with 10 mM sodium acetate. The process was carried out until the volume of the retentate with highly active enzyme was reduced 20-fold.

#### Final purification/chromatography

The enzyme was further purified with the use of fast protein liquid chromatography (FPLC) employing ÄKTA 25 system (GE Healthcare Life Sciences) and a Q Resource column (30 × 6.4 mm; GE Healthcare Life Sciences). Proteins were eluted with a linear gradient of 0–0.2 M NaCl in 10 mM sodium acetate buffer (pH 5.0) at a flow rate of 1 mL/min. Fractions containing laccase activity were found in two major peaks designated as Isoform I and Isoform II. They were separately collected and concentrated as well as dialyzed with centrifugal filter units (5 kDa, Sartorius, United Kingdom) and stored at 4 °C.

### Analytical methods/procedures

Laccase activity was determined by measuring the oxidation of 500 µM 2,2′-azino-bis(3-ethylthiazoline-6-sulfonate) (ABTS) buffered with 50 mM citrate–phosphate buffer (pH 4.5, *ε*_420_ = 36 1/(mM cm)). All spectrophotometric measurements were carried out using a UV/VIS T80^+^ spectrophotometer (PG Instruments Ltd.). Enzyme activities were expressed in catalytic units defined as 1 µmol of product formed per min.

Glucose concentration was determined with the use of liquid chromatography (UPLC^®^, Aquity Waters, USA) and detected with the evaporative light scattering detector. Aquity BEH Amide chromatographic column was thermostated at 35 °C and the mobile phase flow rate was equal to 0.29 mL/min. Elution was carried out with 75% CH_3_CN supplemented with 1% triethylamine. Under these analytical conditions, glucose was eluted at the retention time of 4.47 min. The accuracy of the determination was ± 0.02 g/L.

Biomass concentration was determined as dry weight according to a following procedure: 40 mL of suspension was filtered under atmospheric pressure through a paper filter (Filtrak 389) of the predetermined mass. The filter with biomass was dried at 105 °C and weighed after 24 h. The accuracy of the determination of biomass concentration was ± 0.4 g/L.

Molecular weight (MW) of the laccase isoforms was determined by sodium dodecyl sulfate–polyacrylamide gel electrophoresis (SDS-PAGE) using the Bolt Mini Gel Tank and Bolt Bis–Tris Plus 4 12% gels (Thermo Fisher Scientific). Bolt MES SDS served as the running buffer. SeeBlue Plus2 Pre-stained Protein Standard (Thermo Fisher Scientific) was used as a molecular weight standard. The analytic procedure was performed according to the manufacturer’s instructions with sample volume of 26 µL. Protein samples were then reduced with the use of Bolt Reducing Agent (Thermo Fisher Scientific). SimplyBlue SafeStain (Thermo Fisher Scientific) was applied as the protein stain.

Isoelectric points (p*I*s) were estimated using the XCell SureLock Mini-Cell Electrophoresis System and Novex pH 3-7 IEF Protein Gels (Thermo Fisher Scientific). SERVA Liquid Mix IEF 3-10 Marker served as the protein standard. The analyses were performed according to the manufacturer’s recommendations with sample volume of 5 µL. Following isoelectric focusing, the gels were fixed in 12% trichloroacetic acid for 30 min and subsequently stained with SimplyBlue SafeStain (Thermo Fisher Scientific).

Protein concentration was determined with the use of Bicinchoninic Acid Kit (Sigma-Aldrich) according to the manufacturer’s instructions with sample volume of 25 µL. The measurements were performed using a T80^+^ UV/Vis Spectrometer (PG Instruments Ltd).

UV–Vis absorption spectra of purified laccases were recorded in 10 mM Na acetate buffer (pH 5) in the range from 250 to 800 nm using a T80^+^ UV/Vis Spectrometer (PG Instruments Ltd). All chemicals were obtained from Sigma-Aldrich (Steinheim, Germany) and Merck.

### Calculated parameters

There are two main parameters which determine the efficiency of the purification process: the activity recovery yield (yield), which informs about the recovery of the enzyme in a given method and the purification fold, which refers to the number of times a protein preparation is enriched for the protein being purified. The yield was calculated as the ratio of final total laccase activity (after the specific purification step) to initial total laccase activity (before the specific purification step), where the total activity in units (U) is the volume activity (U/L) multiplied by volume of the given phase.

$${\text{Yield}}\;\left( \% \right) = \frac{{{\text{final}}\;{\text{total}}\,{\text{activity}}}}{{{\text{initial}}\,{\text{total}}\,{\text{activity}}}} \times 100$$

The purification fold was calculated as the ratio of final specific activity of laccase after the given purification step to initial specific activity of laccase before the purification step. The specific activity is the ratio of protein total activity to protein content (U/mg).

$${\text{Purification}}\,{\text{fold}} = \frac{{{\text{final}}\,{\text{specific}}\,{\text{activity}}}}{{{\text{initial}}\,{\text{specific}}\,{\text{activity}}}}$$

where the *final* refers to the phase with concentrated laccase (after the specific purification step), and the *initial* refers to the activity of the phase before the specific purification step.

## Results

In the present study, the optimal system for laccase production and purification as well as comparison of the three different downstream processing methods was investigated. The experimental work involved bioreactor cultivation of fungus *Cerrena unicolor* for laccase biosynthesis and then the enzyme separation from fermentation broth and concentration or purification. The purification procedure involved, as the first step, aqueous two-phase extraction, foam fractionation, ultrafiltration and the fast protein liquid chromatography at the end. The obtained products (isoforms) were separately pooled and partially characterized.

### Cultivation of *Cerrena unicolor*

**Fig. 2:**
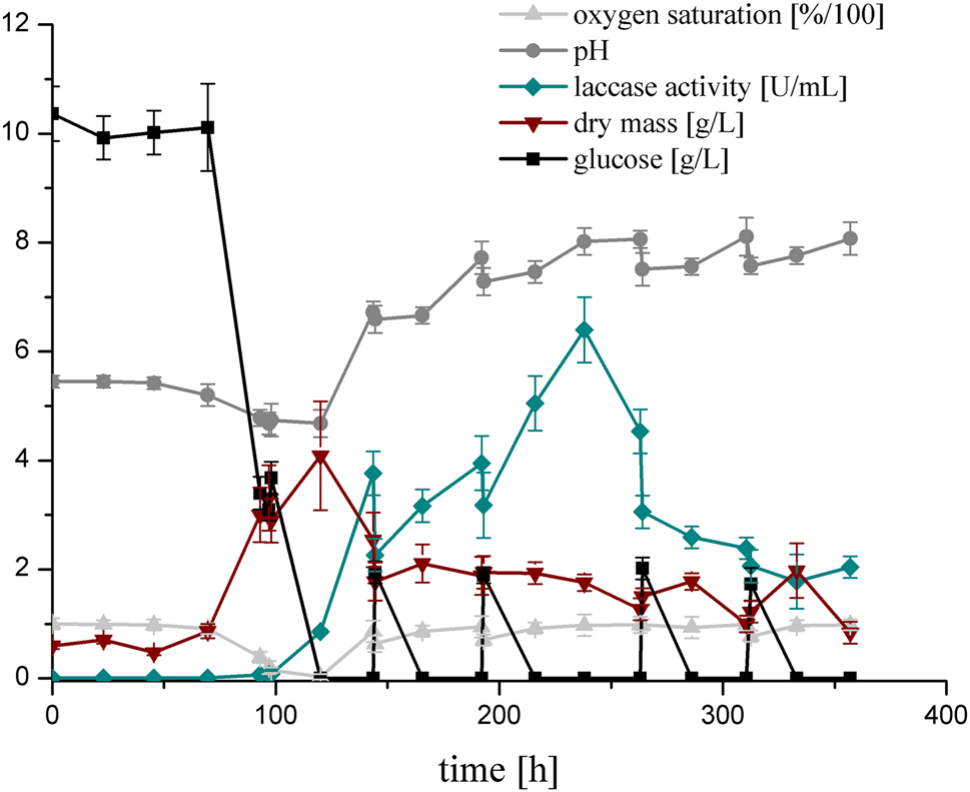
Time course of glucose, biomass, laccase activity, oxygen saturation and pH of the medium during *Cerrena unicolor* cultivation in bioreactor; the presented points represent the averaged values from three experiments and error bars are standard deviation

The time course of cultivation parameters: substrate utilization, biomass growth and laccase activity, as well as changes in pH and oxygen saturation of the medium are presented in Fig. 2.

At the beginning of the process, a significant lag phase was observed, which is typical for this fungal strain [28]. Then, in the trophophase, glucose utilization began and the exponential growth of biomass was observed. The moment when glucose was ultimately used up was accompanied by the maximum biomass concentration and corresponded to the beginning of laccase secretion. The decrease in oxygen saturation and pH value was also observed. Starting from day 6 (144 h) of the process, the addition of the medium was applied every second day. This kind of repeated fed-batch mode resulted in high laccase activity (up to 4 U/mL) from day 6 to day 9 which remained stable owing to regular refills with fresh medium. After 11 days of fungal growth, the cultures were harvested and laccase activity reached its maximum of 6.39 U/mL. Afterwards, regardless of fresh batches of nutrients added to the system, the laccase activity decreased. This could be caused by the excess of biomass remaining in the bioreactor, which prevented its further growth. Interestingly, the prolonged production of laccase was accompanied by stable oxygen saturation and a considerable increase in pH from 4.5 to 8.5. This observation was in agreement with earlier findings, as the pH increase was previously recorded in laccase-oriented cultivations of *C. unicolor* [29].

### Comparison of downstream processing methods of laccase

After the filtration of the culture liquid from a bioreactor, the crude enzyme extract with an average activity of 7.295 U/mL was achieved. Next, three methods were applied to compare their efficiency and further utility.

#### Aqueous two-phase extraction

Aqueous two-phase extraction approach was used under the conditions described in previous study [16]. The obtained results concerning the phase volume, laccase activity and protein content, as well as calculated activity yields and purification folds are presented in Table 1.

**Table 1:** The results for aqueous two-phase extraction of laccase

	Volume (mL)	Activity (U/mL)	Protein (mg)	Yield (%)	Purification fold
Culture liquid	40.8 ± 0.1	7.295 ± 0.07	1.46 ± 0.02	100	1
Salt phase	40 ± 0.4	7.250 ± 0.36	0.34 ± 0.02	97.4	4.2
PEG phase	39.2 ± 0.4	0.066 ± 0.003	0.26 ± 0.01	0.9	0.05

According to the results, the laccase was concentrated in the salt phase. From 40.8 mL of crude enzyme extract with an activity of 7.295 U/mL, 40 mL of a salt phase with a laccase activity of 7.25 U/mL and 39.2 mL of a polymer phase with an insignificant activity of 0.066 U/mL were obtained, which means that from 297.6 U of initial total activity, 290 U went to a salt phase and only 2.58 U went to polymer phase. It shows also that the loss of enzyme activity was very low, about 1.7% (5.02 U). This confirmed that ATPE allows for reaching of high laccase recovery. After the extraction, 97.4% of laccase was found in the salt phase and the purification fold was equal to 4.2.

#### Foam fractionation

As the second purification method, foam fractionation was performed under the earlier determined conditions [25]. The results concerning the phase volume, laccase activity and protein content, as well as the calculated activity yields and purification folds are presented in Table 2.

**Table 2:** The results for foam fractionation of laccase

	Volume (mL)	Activity (U/mL)	Protein (mg)	Yield (%)	Purification fold
Culture liquid	100 ± 0.5	7.295 ± 0.07	3.57 ± 0.04	100	1
Foamate	6.1 ± 0.3	29.8 ± 1.5	0.63 ± 0.03	24.9	1.4
Retentate	88 ± 4.4	2.9 ± 0.16	2.45 ± 0.12	35	0.5

After the process was completed, from each 100 mL of culture fluid with an activity of 7.295 U/mL (total activity of 729.5 U), 6.1 mL of foamate with an activity of 29.8 U/mL (total activity of 182 U) was obtained. It turned out that a retentate with the volume of 88 mL still contained laccase with the activity of 2.9 U/mL, which means 255 U. However, the concentration effect did occur as in the foamate the laccase activity was ten times higher than in the retentate (29.8 U/mL and 2.9 U/mL, respectively). Despite the lower recovery yield of foamate, the purification fold of this phase was almost three times higher than the one recorded for the retentate. However, as a result of the process, almost 40% of the laccase activity was lost. This may be caused by the addition of the cationic surfactant, namely CTAB. As described by Azimi et al. [30], CTAB can cause a decrease in laccase activity, but, on the other hand, it is also necessary to achieve good foaming effect. Linke et al. [23] tested a number of surfactants and selected CTAB as the most promising compound with regard to process improvement.

#### Ultrafiltration

In addition to the two above-described methods, the traditional ultrafiltration with 10 and 30 kDa cutoff membranes was applied (Table 3). In the first step, involving the membrane with the cut-off value of 10 kDa, 1 L of crude laccase extract with the total activity of 7295 U was concentrated 20-fold. The obtained retentate with the volume of 50 mL had the total laccase activity of 7112 U. Hence, the recovery yield of 97.5% was achieved with the purification fold of 4.6. Then, the retentate was diluted to a final volume of 1000 mL and passed through the second step of ultrafiltration with the membrane of 30 kDa cut-off point. These processes caused the reduction of volume back to 50 mL. However, in this case, the total activity of retentate was 5379 U, which leads to the total yield of two-step ultrafiltration equal to 73.7%. However, the purification fold was then higher and equal to 6.6.

**Table 3:** Purification of laccase from *Cerrena unicolor* using three different methods

	Volume (mL)	Protein (mg)	Total activity (U)	Specific activity (U/mg)	Yield (%)	Purification fold
Culture liquid	1000	35.74	7295	204.1	100	1
Ultrafiltration cut-off 10 kDa	50	7.63	7112	932.1	97.5	4.6
Ultrafiltration cut-off 10, 30 kDa	50	3.98	5379	1352	73.7	6.6
Foam fractionation	61	6.31	1818	288.1	24.9	1.4
ATPE	980	8.33	7105	852.9	97.4	4.2

The final results concerning yields and purification folds calculated for 1 L of crude laccase extract for the three methods used are presented in Table 3.

Based on the purification fold values presented in Table 3, it could be stated that the best results were obtained for two-stage ultrafiltration using membranes with the cut-off values of 10 and 30 kDa, where the purification fold was equal to 6.6. During foam fractionation, the enzyme in foamate was concentrated, but no significant separation of laccase from other proteins was obtained, and the purification fold had the lowest value of 1.4. While ATPE gave almost the same results as ultrafiltration after the first step with 10 kDa cut-off point, more than fourfold purification was achieved in both cases. As far as the recovery yield is concerned, the measured values were significantly lower for foam fractionation than for other tested methods. For ultrafiltration involving a 10 kDa cut-off membrane and ATPE the recovery yield was very high—above 97%. A slightly lower recovery yield value of 73.7% was achieved for two-stage ultrafiltration.

### Final purification of laccase

**Fig. 3:**
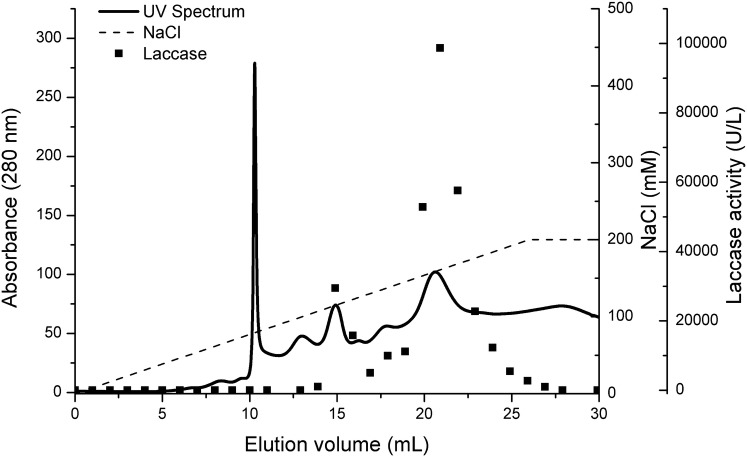
Purification of *Cerrena unicolor* laccase by anion exchange chromatography

For the final purification of laccase, the retentate collected after two-step ultrafiltration was subjected to the fast protein liquid chromatography with a Q Resource column. That choice was related to the fact that the laccase extract after ATPE would require additional treatment. After only one chromatographic step, two distinct fractions with laccase activity were obtained as presented in Fig. 3. They were separately collected, centrifuged to remove remaining eluent and stored at 4 °C for further analysis.

After chromatographic analyses, fractions containing laccase were found in two major peaks (elution volume at 15 and 21 mL) interpreted as two isoforms, however, two more indistinct peaks of laccase could be noticed at elution volume 13 and 18 mL. The two main isoforms of specific activity of 2349 and 3374 U/mg were taken into consideration. The results of the complete process/procedure of laccase purification to apparent homogeneity are summarized in Table 4.

**Table 4:** Purification of *Cerrena unicolor* laccase

Purification step	Volume (mL)	Protein (mg)	Total activity (U)	Specific activity (U/mg)	Yield (%)	Purification fold
Culture liquid	1000	35.74	7295	204.1	100	1
Ultrafiltration cutoff 10, 30 kDa	50	3.98	5379	1352	73.7	6.6
Isoform I Resource Q	1.812	0.175	411	2349	5.63	11.51
Isoform II Resource Q	2.234	0.516	1741	3374	23.87	16.53

**Fig. 4:**
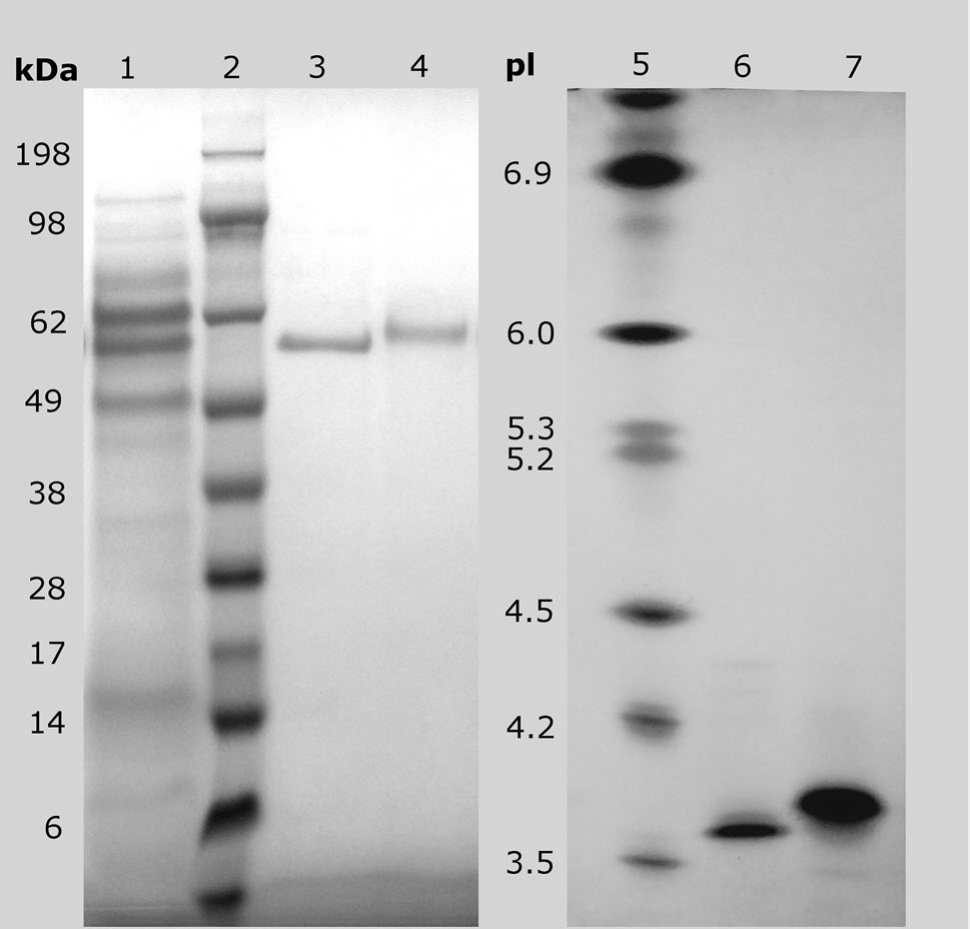
SDS-PAGE (left) and isoelectric focusing (right) of purified *Cerrena unicolor* laccase isoforms. Lanes: 1—crude laccase, 2—protein standards, 3—Isoform I, 4—Isoform II, 5—protein standards, 6—Isoform I, 7—Isoform II

The final volumes of the fractions were 1.812 mL and 2.234 mL with the total activity of 411 U and 1741 U, respectively. Both fractions had a bluish tinge, which was visible on the spectrum (data not shown). The molecular masses of the fractions were approximately 57 and 64 kDa (Fig. 3), which confirms the previous results of Michniewicz et al. [29]. As depicted in Fig. 4, the p*I* values of both laccase isoforms were about 3.6 and 3.7, respectively.

## Discussion

The repeated fed-batch mode applied for *Cerrena unicolor* cultivation proved to be very efficient towards the production of highly active laccase. The maximal activity of 6400 U/L was about four times higher than previously recorded for the conventional batch bioreactor cultivation [16] and for the shake flaks cultures of this fungus [25]. The achieved activity is also satisfactory in comparison to the values reported in the literature obtained for other fungal strains: 30.2 U/L for *Phanerochaete chrysosporium* during batch fermentation [31], 1403 U/L for *Pleurotus ostreatus* in packed-bed bioreactor [32] or 2123 U/L for *Trametes versicolor* in fluidized-bed bioreactor [33]. What is more, due to the constant feeding with fresh medium, it was possible to maintain a high enzyme activity for a long time. Based on the information that the biosynthesis of laccase is influenced by the culture medium composition, especially with respect to the amount of carbon [28], it was proved that the addition of small amounts of medium keeps the fungus active and allows to maintain the biosynthesis of laccase in the culture. Therefore, it was possible to prolong high activity of the supernatant compared to a typical batch culture, where normally after distinct maximum a fast decrease in activity is observed. In this case, the maximum was obtained after the second refill. The further decrease in activity (after its distinct maximum) could be caused by an excessive amount of biomass remaining in the bioreactor, which prevented its further growth. On the other hand, the use of a rotary spin filter enabled the collection of culture liquid without biomass particles. As a result, the further purification process was shortened by the step of biomass filtration in situ. Moreover, the applied system enabled to integrate the processes of biosynthesis and filtration of laccase within a single apparatus.

Subsequently, laccase with the highest activity was subjected to two unconventional methods of concentration and purification: aqueous two-phase extraction and foam fractionation. The studies on the application and comparison of these downstream processing methods for a single batch of crude laccase extract, to the best of our knowledge, have not been published before. These methods have been tested, but always separately for laccases from various sources and with different activities or properties. Therefore, it was difficult to compare their efficiency and effectiveness. The results of the present study open the door for such comparisons. Concerning aqueous two-phase extraction, our results confirm an earlier observation that laccase from *C. unicolor* is concentrated in a salt phase while using PEG 6000 and phosphate system. The relatively high recovery yield of 97.4% is comparable with the yield recorded after first step of ultrafiltration with the membrane of 10 kDa cutoff equal to 97.5%. The recovery yield achieved in the present study lies well within the range or even exceeds the values reached by other researchers using ATPS for purification of fungal laccases. Ratanapongleka [34] obtained the 99.08% yield of enzyme activity when applying PEG 4000 and potassium phosphate system at pH 7.0. Prinz et al. [35] and Mayolo-Deloisa et al. [36] also proved that PEG-phosphate aqueous two-phase system is suitable for the primary recovery of laccase. In the first case [35], PEG 3000-phosphate was successfully used with the purification factor of 2.74 and activity recovery of 96%, while in the second case [36] PEG 1000-phosphate ATPS resulted in an overall yield of 95% in a single-stage recovery process. However, in all cited systems, the enzyme partitioned in the top polymer-rich phase, in contrast to the results reported in this work, where laccase from *C. unicolor* in PEG 6000-salt systems tended to partition towards the bottom salt-rich phase. From a practical point of view, such behavior is desired due to the simplicity of further purification steps.

The foam fractionation was carried out in an apparatus which facilitated steady-state conditions. However, the achieved concentration of the product in the foamate phase was not as satisfactory as the ATPE. Linke et al. [23] applied foam fractionation for commercially available pure laccase from *Trametes* sp. They achieved very good results (recovery of 94% at pH 6.0), however, in these experiments powdered laccase dissolved in deionized water was used. The enzyme from the fermentation broth was less susceptible to partition under the same conditions, only 24% of the laccase activity accumulated in the foam, while 74% remained in the retentate. Only after changing the process conditions and increasing the detergent concentration, the results were improved [23]. Therefore, because of the lowest level of purification fold (1.4), foam fractionation seems to be the least attractive alternative. However, as it is a gentle, inexpensive and environmentally compatible method, it can be applied as the initial step in the whole purification process.

For the final chromatographic purification of *Cerrena unicolor* laccase, the enzyme solution concentrated by two-step ultrafiltration, with purification fold of 6.6, was applied. Although the recovery yield was higher after ATPE, ultrafiltration was more desirable, since the subsequent enzyme purification was easier and led to lower activity losses compared to the ATPE system. In the studied conditions after chromatographic analyses, fractions containing laccase were found in four peaks, wherein only two major peaks interpreted as two isoforms with specific laccase activity of 2349 and 3374 U/mg were further examined. The obtained result confirmed earlier investigation results described by Michniewicz et al. [29], but this time the specific activity of both isoforms was about 4.8 and 16 times higher, respectively. Again, another two isoforms which were noticed and described by Rogalski and Janusz [37] were not separated, because of their very low activity titer. A thermostable laccase from marine-derived fungus MTCC 5159 identified as *C. unicolor* produced three distinct laccase isoforms, but only one that had clearly higher activity was purified and characterized [38]. In our study, the specific activity of the culture liquid was about 13 and 4000 times higher than previously reported by Kim et al. [39] and Gianfreda et al. [40], respectively. The whole process of purification consisted of only two stages, after which a high recovery yield of 23.9% and purification factor of 16.5 for Isoform II was achieved. Similar results for *C. unicolor* were described previously, but the corresponding purification procedures comprised a greater number of stages, namely five stages of purification with three chromatographic steps, as reported by Kim et al. [39], or four stages of purification in the study of Wang et al. [41]. Yang et al. [42], who studied the laccase derived from *Cerrena* sp. HYB07, obtained three times higher specific activity of laccase in culture supernatant, but after the purification procedure, the purification factor was only 3.1. The obtained p*I* of about 3.6 and MW of 57 and 64 kDa lie well within the range determined for other fungal laccases [43].

**Fig. 5:**
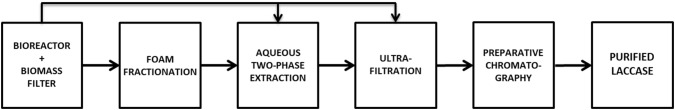
Proposed sequences of concentration and separation of laccase from a culture liquid

To sum up, it can be stated that the conducted research can be treated as groundwork for designing a compact integrated system for the production, concentration and separation of fungal laccases, as presented in the figure below (Fig. 5):

Biosynthesis of the enzyme is carried out in a bioreactor with a spin filter in the repeated fed-batch mode. The obtained supernatant is initially concentrated by foam fractionation or/and extracted in a polyethylene glycol–phosphate aqueous two-phase system. The laccase-rich extract is then dialyzed via ultrafiltration and finally purified by preparative liquid chromatography. In a shortened, but less economical, version of the procedure, the enzyme is subjected solely to ultrafiltration and chromatographic purification. In all cases, the final product, displaying high purity and enzymatic activity, can be utilized in various industries, including, but not limited to, paper, textile and food industries.

## Conclusions

1. Repeated fed-batch mode proved to be successful in terms of increasing laccase production in *Cerrena unicolor* cultures;
2. Aqueous two-phase extraction can be successfully applied for laccase purification;
3. ATPE in PEG 6000-phosphate system allows for a high recovery yield of 97.4% and purification factor of 4.2. In turn, although FF generates similar losses with respect to laccase activity, it has many advantages (low cost) and can be applied when the partial concentration is sufficient;
4. A relatively easy and fast two-step procedure of laccase purification was proposed. The suggested approach led to enzyme recovery yield of 23.9% and purification fold of 16.5;
5. The two main laccase isoforms with high specific activities of 2349 and 3374 U/mg were obtained;
6. The results obtained could be the basis of the integrated system for the biosynthesis and separation, concentration and purification which can be applied not only for laccase but also for other biomolecules, e.g., enzymes, viruses, nucleic acids and monoclonal antibodies.
